# Effects of the Storage Conditions on the Stability of Natural and Synthetic Cannabis in Biological Matrices for Forensic Toxicology Analysis: An Update from the Literature

**DOI:** 10.3390/metabo12090801

**Published:** 2022-08-27

**Authors:** Elias Djilali, Lucia Pappalardo, Anna Maria Posadino, Roberta Giordo, Gianfranco Pintus

**Affiliations:** 1 Department of Biology, Chemistry and Environmental Sciences, American University of Sharjah, Sharjah P.O. Box 26666, United Arab Emirates; 2 Department of Biomedical Sciences, University of Sassari, 07100 Sassari, Italy; 3 College of Medicine, Mohammed Bin Rashid University of Medicine and Health Sciences, Dubai P.O. Box 505055, United Arab Emirates

**Keywords:** cannabinoids, stability, urine, plasma, oral fluids, hair, dried blood spots

## Abstract

The use and abuse of cannabis, be it for medicinal or recreational purposes, is widely spread among the population. Consequently, a market for more potent and consequently more toxic synthetic cannabinoids has flourished, and with it, the need for accurate testing of these substances in intoxicated people. In this regard, one of the critical factors in forensic toxicology is the stability of these drugs in different biological matrices due to different storage conditions. This review aims to present the most updated and relevant literature of studies performed on the effects of different storage conditions on the stability of cannabis compounds present in various biological matrices, such as blood and plasma, urine, and oral fluids, as well as in alternative matrices, such as breath, bile fluid, hair, sweat, cerumen, and dried blood spots.

## 1. Introduction

As of 2020, cannabis has become the most frequently used drug worldwide. Its use is associated with the impairment of the assuming individual’s cognitive and psychomotor abilities [1]. However, authentic marijuana is not the sole cause of concern, as synthetic cannabinoids also exist. These drugs were created to mimic the binding of Delta-9-Tetrahydrocannabinol (THC) to the Cannabinoid Receptor 1 (CB1) and Cannabinoid Receptor 2 (CB2). However, it was later discovered that the binding potential possessed by these synthetic drugs is far more strong than that of their natural counterparts, causing them to have a greater chance of resulting in toxic effects [2]. Most of the abused synthetic cannabinoids legally available on the market appear to be CB1 receptor agonists showing an affinity greater than THC [3]. Due to their stronger cannabimimetic effects, a greater incidence of cognitive and psychomotor impairment, seizures, psychosis, tissue injury, and death associated with these drugs’ intake has been observed [4]. Data have shown that accidents, sometimes resulting in fatalities, have grown in number due to the increased use of these drugs [1]. The primary psychoactive components of cannabis are THC and its metabolites, primarily THCCOOH. As a consequence, given the increment of both the use and abuse of such psychoactive substances, it is imperative for forensic laboratories to properly understand their stability within the biological matrices of collection. Indeed, their degradation is one of the most significant causes of concern during forensic cases [5]. These compounds are, in fact, subject to numerous processes that lead to the eventual degradation of or decrease in the cannabinoids from the sample. Such processes include but are not limited to conjugate formation, adsorption to surface containers, microbial action, thermal decomposition, and sample handling errors [6,7]. Therefore, sample storage conditions are critical for forensic toxicology analysis. This review will provide insights into the overall stability of cannabinoids within different conventional and alternative biological matrices, namely blood, plasma, urine, oral fluids, breath, bile fluid, hair, sweat, cerumen, and dried blood spots, and gather the currently published literature about the ideal sample storage conditions for forensic toxicology analysis.

## 2. Conventional Biological Matrices

### 2.1. Blood and Plasma

Analyte stability is among the essential parameters in forensic toxicology [8]. In blood, THC concentration reaches its highest point approximately 10 min after smoking cannabis and is then quickly distributed throughout the body due to its lipophilic nature. THCCOOH, its metabolite, on the other hand, can persist within the body for up to a month [1]. Therefore, studies of these two metabolites have become more prominent in the past decade, as they may provide a practical guideline to properly detect the abuse of cannabinoids in forensic cases. To better understand the stability of these cannabinoids, different storage temperatures (room temperature, refrigerated, and frozen) over time were carefully examined, since the concentration of both THC and THCCOOH is time-dependent [9]. Where the temperature is concerned, storing blood samples containing cannabinoids in a frozen condition, or refrigerated at the very least, appears to be the most effective way to ensure the greatest stability for the longest period of time [10]. At room temperature, cannabinoid concentrations tend to significantly decrease after a time ranging between two weeks and two months, regardless of the container material [10]. Storing whole blood containing cannabinoids in Venoject tubes with rubber stoppers for 6 months at room temperature decreased their concentrations by approximately 90%. Johnson et al. highlight the possibility of a THC concentration loss to the rubber stoppers used for the containers, but no further data is provided [10].

Furthermore, other variables to consider are the properties of the containers in which the matrices are being stored. Because of the cannabinoids’ lipophilic nature, studies have highlighted the possibility of a drug adsorptive loss onto the container, which is made of similarly lipophilic plastic [11]. Experimental studies comparing the efficacy of polystyrene plastic and glass vials on THC-containing whole blood samples stored at −20 °C for 4–24 weeks showed a loss of THC concentration of 60 to 100% in the samples stored in plastic containers, while a loss of 30 to 50% was observed in the samples stored in glass vials [11].

Whole-blood-contained cannabinoids stored in green-top sodium heparin vacutainers were found to remain stable for 3–4 months when stored under refrigerated conditions, whereas when stored under frozen conditions, they remained stable for up to 6 months [12]. The same tests were executed on plasma samples stored in grey-top sodium fluoride tubes, with results showing that cannabinoids would remain stable for up to 12 months at −20 °C [13]. However, it is worth mentioning that the same results were not observed in all the THC metabolites. Toennes and Kauert reported that, in plasma, the THCCOOH ester glucuronide metabolite, called THCCOOH-glucoronide (THCCOOH-glu), tends to significantly degrade. The study concluded that the susceptibility of the metabolite to the esterase enzymes naturally present in the blood might be at the base of the observed phenomenon [14]. Fort et al. performed a similar experiment on synthetic cannabinoids, namely XLR-11, UR-144, AB-Pinaca, and AB-Fubinaca, obtaining similar results [2]. The concentration of the synthetic cannabinoids was stable for the entire period of the experiment (12 weeks) when the blood samples were kept frozen. In contrast, under the other two conditions (refrigerated and room temperature), there was a significant loss of the samples spiked with XLR-11, while the concentrations of UR-144, AB-Pinaca, and AB-Fubinaca remained stable at all three different temperatures for the entire experiment duration (t = 12 weeks) [2]. Similarly to THCCOOH-glu, AB-Pinaca and AB-Fubinaca were found to be susceptible to degradation by carboxylesterase enzymes [4]. WIN 55,212-2 is another synthetic cannabinoid that was observed to be metabolized by the hepatic microsomes at the same rates as the previously mentioned synthetic cannabinoids. Its metabolites may be extracted for detection purposes from bio-matrices, although further research is required to fully confirm this aspect [15].

Using whole blood samples collected in glass vials, Meneses and Mata repeated similar experiments on different cannabis compounds, namely 11-nor-9-carboxy-THC, Cannabinol, and Cannabidiol under refrigerated and frozen conditions. The study results showed that the cannabinoids remained stable for approximately 6 months, losing about 20% of their initial concentration. While working with samples suspected of containing cannabinoids, the authors concluded that it would be ideal to analyze the samples as rapidly as possible, as it would provide the most accurate results. Should that not be possible, storage under frozen conditions is recommended [16]. Hess et al. analyzed the freeze/thaw stability of several synthetic cannabinoids in glass tubes, concluding that, while not advisable, continuously freezing and thawing a serum sample containing synthetic cannabinoids does not significantly decrease the initial drugs’ concentration [17]. On the other hand, another study performed on whole blood stored at −20 °C in plastic vacuette containers observed a significant difference between samples that had undergone freeze/thaw multiple times and samples that remained frozen uninterruptedly. This study, however, showed that the decrease in stability and concentration over time can be avoided using anti-oxidants as preserving agents. Indeed, applying a mixture of Fluoride Oxalate (FX) and Ascorbic acid (ASC) to the samples resulted in no significant cannabinoid loss after 5 months, even when storage was interrupted by six freeze/thaw cycles [18]. A summary of the reported data is presented in Table 1 and Table 2.

### 2.2. Urine

Urine, among the other biological matrices used for illicit drug detection, is considered the most popular. Indeed, urine sapling requires noninvasive collection techniques and allows for a fairly wide detection window for most psychoactive drugs and their metabolites [19]. Due to its easy application, urine drug testing is often used in workplaces to test all workers to create a ‘drug-free work environment’ [20]. Thus, similarly to blood, a thorough understanding of the drugs of abuse stability in urine matrices is essential. In urine, the stability of a drug depends on the sample pH, storage temperature, bacterial contamination, and the container material used [21]. In this context, Ciuti et al. tested the effects of temperature (−20 °C, 4 °C, and 25 °C), over 20 weeks on THC-containing urine samples using both glass and polyethylene vials. The data indicated a recovery of approximately 85% of the original content in samples stored at −20 °C (frozen conditions), thus indicating the analytes’ relative stability. Conversely, this was not observed in samples stored at 4 °C and at 25 °C, where the recovery was 37 and 33%, respectively [21]. These findings align with another study’s conclusions, whereby frozen conditions allowed for greater cannabinoid stability within the urine matrix. This experimental study spanned over 3 years and showed a maximum loss in cannabinoid (THCCOOH) concentration of 19.6 ± 6.7% when samples were stored at −20 °C in polypropylene containers [22]. Desrosiers et al. replicated a similar experimental design and were also able to observe better cannabinoid stability when samples were stored under frozen conditions for up to 6 months. In their experiment, polypropylene vials were utilized instead of polyethylene ones as they seemingly cause less adsorptive loss [23]. The authors also stated that glass vials are less preferred to store biological matrices due to the easy possibility of breaking [23]. Frozen conditions appear to be the most favorable for another THC conjugate, THCCOOH glucuronide, a THCCOOH metabolite. Unlike in blood, however, the THCCOOH-Glu-degrading esterase enzymes are not present in urine, allowing this molecule to remain present within the solution, and therefore making it a viable marker for the detection of cannabis use [7].

Further insight on the cannabinoids’ stability has been provided by studies focusing on the containers utilized to store the drugs of abuse. Jamerson et al. showed the effects of container composition, pH, and temperature on the cannabinoids’ adsorptive loss. Tests performed using polypropylene plastic containers and borosilicate glass containers showed that the adsorptive loss was highly present in polypropylene containers compared to the borosilicate ones and that it appeared to be relatively absent in urine solutions near neutral or basic pH [24]. Although glass vials show no cannabinoid adsorptive loss, their usage is not the preferred one when it comes to the storage of biological matrices due to easy breakability [23]. In light of such conclusions, researchers have tried to observe whether the type of plastic container employed may cause lower, or higher, cannabinoid metabolite adsorptive loss [25]. In this regard, the effect of both polypropylene and polyethylene containers on cannabinoid stability was tested at both 4 °C and 25 °C in the same study. A rapid cannabinoid loss was observed for both containers at 4 °C, while at 25 °C only a small loss was observed for polypropylene containers, and no significant loss was observed in polyethylene containers. The authors mentioned that the observed effects could be related to the cannabinoid’s lower solubility in water at lower temperatures. In addition, as the overall loss appeared to stabilize after approximately 1 h, the researchers concluded that the observed loss was due to a surface phenomenon and not to an absorption effect into the container plastic matrix [25]. Similarly, it was determined that a solution of urine spiked with THC could be stored in (Nalgene^®^) high-density polyethylene plastic containers for up to 40 days. The study illustrated that, at 2–8 °C, the analyte concentration remained constant for 42 days and showed a minimal decrease following day 42. The analyte concentration decreased from 72.44 ng/mL to 65.71 ng/mL on day 72 [26]. While trying to understand the mechanism of cannabinoid concentration loss in urine matrix, research studies showed that loss could be divided into loss during equilibrium conditions, that is, during storage, and loss during kinetic conditions, indicating losses that occur while transporting, manipulating, and testing urine samples [27]. The study’s conclusion showed that equilibrium losses are affected by the solvent, the container material, and the exposed surface area. In contrast, kinetic losses are affected mainly by temperature. Furthermore, Roth et al. advised the usage of glass containers for storage and glass pipettes for sample handling. Conversely, the poorest results were observed when using high-density polyethylene containers [27]. Lastly, using containers possessing internal bar code labels is not advised, as test results showed a significant reduction in THCCOOH levels when urine samples were stored in Doxtech bottles with an internal bar code. Instead, losses were relatively insignificant when urine samples were stored in the same containers but with an external barcode instead. This phenomenon appears to be due to the internal ID itself being made of waterproof polypropylene materials [28]. In their study, Welsh et al. reported that the adsorptive loss issue during the sample’s storing and handling might be bypassed if the cannabinoid-containing urine solution is treated with a non-ionic surfactant such as Tergitol. Their results showed a significantly higher THC recovery from the surfactant-treated samples [29]. Additionally, fungal and bacterial growth appear to be factors involved in significantly decreasing cannabinoid concentration in urine samples. However, this decrease appears to occur only when the storage temperature is above a threshold (near room temperature) that would allow for bacterial and fungal growth in the first place [7]. However, it is yet to be determined whether bacteria and fungi possess the ability to specifically degrade cannabinoids or otherwise [5]. A summary of the reported data is presented in Table 3.

### 2.3. Oral Fluids

When it comes to psychoactive impairment caused by cannabinoid drugs, oral fluids have become increasingly studied biological matrices for the early detection of drugs of abuse. The reasons for their increase in popularity are several, including noninvasive collection methods, no requirement for trained medical professionals, and the possibility of multiple sample collection allowing for early detection in workplaces [30]. Reports show that oral fluids can also be utilized for the detection of New Psychoactive Drugs (NPS), such as new synthetic cannabinoids that continuously appear on the market [31].

Just like it has been done for blood and urine, to further understand the cannabinoids’ stability in oral fluids, researchers have studied the effects of storage temperature and sample container material on the psychoactive drug in this biological matrix. When stored at −20 °C, 4 °C, and 21 °C in polypropylene plastic containers for 6 weeks, THC losses were reported to be 21%, 87%, and 86%, respectively [32]. Similar tests were repeated using expectorated oral fluids stored for 6 days in polypropylene tubes and glass tubes at 4 °C and room temperature. Results showed a loss of <10% for the samples stored in glass vials at both temperature conditions and >20% when stored in polypropylene tubes [33]. Kneisel et al. compared the effects of glass and plastic containers on 11 synthetic cannabinoids [34]. After 24 h of storage in polypropylene tubes at room temperature, the authors observed recoveries ranging from 29 to 65%, while at 4 °C, recoveries ranged between 83 and 103% (RSD ≤ 13%). After 72 h of storage in plastic containers, recoveries dropped to a range between 9 and 54% at room temperature and 75–79% at 4 °C. When using RapidEASE borosilicate glass tubes, on the other hand, recoveries ranged between 84 and 114% (RSD ≤ 12%) for the entire duration of the experiment (72 h) and at all temperature conditions [34]. Likewise, to confirm that adsorptive loss is indeed the main storage-associated issue of cannabinoid-containing oral fluids, Molnar et al. observed a 23–30% THC adsorptive loss in polypropylene containers within a 6-day storage period [35]. The study determined that lower oral fluid volumes led to a more significant adsorptive loss of the cannabinoid to the tube’s surfaces. Concerning temperature, the oral fluid samples were stored at both 4 °C and room temperature for 4 weeks and a total cannabinoid concentration loss of 40–50% was observed in both temperature conditions [35]. Among the various suggestions, Moore et al. indicated the QuantisalTM collection device as an efficient THC extraction method from oral fluid samples, as long as certain conditions are satisfied [36]. The conditions specified by the authors are in line with the other previously discussed findings and include: the samples must not be in contact with plastic surfaces, must be frozen or refrigerated, and must be stored in the dark [36]. A summary of the reported data is presented in Table 4.

## 3. Alternative Matrices

A rapid increase in the usage of psychoactive drug abuse has been observed in the past decades. For this reason, scientists have been trying to develop novel methods that allow quicker and more precise analyte detection. In the previous sections of this review, we highlighted the more conventional biological matrices used to detect the presence of psychoactive drugs that generally tend to be blood and/or plasma, urine, and, as of lately, oral fluid. As mentioned within this review, each has its advantages and disadvantages. For example, while analysis of cannabinoids present in blood and plasma is very common, the concentrations of the drugs to be determined can, at times, be low and available for short periods [37]. However, as time progresses and improvements are made, new synthetic drugs with similar, albeit more potent, effects are being released on the market [4]. Therefore, it is essential for the scope of the completeness of this review to look at the direction in which researchers are moving towards, that is, developing less invasive and less time-consuming methods [30]. As such, this section will cover the more unconventional matrices that scientists are currently investigating.

Some alternative matrices that could be used to detect cannabinoids in people’s bodies after their consumption have been reported. Among these alternative matrices are breath, bile fluid, hair, sweat, cerumen, and dried blood spots (DBS) [37,38]. As exhaled breath is very commonly used by the authorities to detect alcohol exposure, there is a possibility that it may be used to detect the presence of THC and THCCOOH in exhaled breath following cannabis smoking. Tests showed that THC could be detected from breath between 12 min and 12 h after smoking [39]. Further experiments on 13 chronic smokers showed that the only detectable cannabinoid in breath was THC, while THCCOOH was never detected. In the investigation, all participants resulted THC-positive at 0.89 h after smoking, 76.9% resulted positive after 1.38 h, 53.8% resulted positive at 2.38 h, and only 1 sample out of 13 was positive at 4.2 h [40]. THC concentrations appeared to be higher in the exhaled breath of females rather than that of males. It was concluded that, although much knowledge is still lacking, exhaled breath may be an effective tool in the early detection of THC following cannabis smoke. The authors also highlight the importance of follow-up experiments on the subjects, as the concentrations obtained appeared higher than those reported in previous studies [40]. Similar results were obtained by Karschner et al., setting the maximum time to detect THC in exhaled breath to 3 h [41]. Bile fluids are usually analyzed from corpses in instances where no urine samples are available. A postmortem experiment on 38 corpses showed the presence of THC in a total of 18 cases. More studies are desirable, as no further conclusions were drawn upon the usage of bile for cannabinoid detection [42]. Hair analysis is also a well-established method of drug detection in the forensic field, as it is commonly used to detect cocaine, opioids, and several therapeutic drugs [43]. Recent studies have shown that THC and THCCOOH recovery values from hair samples obtained from people following active cannabis smoke were above 87%. It is important to note that the presence of THCCOOH in the samples excludes passive smoke as a cause for the results. This is because THCCOOH can be formed solely within the body [44]. Another work mentions that hair samples also provide a larger detection window and note that, for the same reasons mentioned above, when using hair samples, it is necessary to monitor THCCOOH rather than THC [41]. Hudson et al. collected fingerprint sweat samples using patches generally placed on individuals’ arms and/or back. The screening cartridge developed in the study was able to detect the drug present in the fingerprint sweat [45]. After comparing these results to those obtained using blood samples, the calculated accuracy of the test reached 96% for THC detection [45].

As it is already known that drugs can be detected in both sebum and sweat, researchers have outlined the possibility that drug detection may also be possible in cerumen, or earwax, as it is a mixture of the two previously mentioned bodily secretions [46]. However, an experiment that tested this hypothesis on 18 subjects comprised of cannabis users provided positive results in only one of the samples. In order to properly determine the validity of cerumen as a tool for cannabinoid detection, however, further research will be required [42]. Concerning DBS, Kyriakou et al., reported data obtained using ultra-high-pressure liquid chromatography tandem mass spectrometry. The analytical recovery for Δ-9-THC, THC-OH, and THC-COOH was 81.1, 79.0, and 78.3. Based on the authors’ data, no relevant analyte instability was observed after maintaining the drug-fortified (50 ng/mL) DBS at room temperature for two weeks. However, in discordance with the urine immunoassay positive results for TCH, when 10 DPS of individuals with acute intoxication were analyzed, only traces of Δ-9-THC and its metabolites could be found in samples 2 and 4 [38]. Consonant with these findings, cannabinoids are among the most challenging analytes in DBS, since, once consumed, they disappear rapidly from blood [47,48]; therefore, their detection in DBS indicates recent intake (within about 2 h) before sampling [49]. Protti et al. tested THC, THC-OH, and THC-COOH stability in six stored, dried DBS over 30 days at RT in regular laboratory storage conditions. DBS were analyzed at different time points (1, 2, 3, 7, 15, and 30 days), giving satisfactory results. Indeed, all the tested analytes fulfilled the acceptance criterion of ±10% assay bias, indicating the compounds’ stability was very good. Moreover, data comparison with plasma cryopreservation demonstrated how DBS could provide increased analyte stability [50]. Mercolini at al. evaluated THC, THC-OH, and THC-COOH stability in blank spiked DBSs stored at room temperature for 1 week, 1 month, or 3 months. Differences in the samples analyzed after spotting and drying were minimal, with a loss of less than 10% even 3 months after sampling. Authors indicated the absence of enzymatic processes due to the drying condition as responsible for maintaining analyte concentration [49].

## 4. Conclusions

The use and abuse of cannabis, be it for medicinal or recreational purposes, has become increasingly widespread. This increment in popularity created, as a result, a market for more potent, and clinically more dangerous, synthetic cannabinoids. As such, it follows that further knowledge on the stability of cannabinoids is required by all fields of science that deal with such substances; critical amongst other factors is a thorough understanding of its stability in different storage conditions, as well as different biological matrices.

Evidence gathered thus far using whole blood or plasma showed that samples should ideally be frozen or refrigerated once collected to ensure better drug stability. Furthermore, whole blood and plasma samples should be collected into glass containers or, at the very least, into plastic vials containing stabilizing agents such as antioxidants. Furthermore, the sample transportation time between collection and analysis and the time the sample spends untreated at room temperature should be reduced as much as possible. It was shown that the mishandling of the sample during transport is also capable of causing a reduction in drug concentration within the matrix.

In urine, traces of cannabinoids or cannabinoid metabolites show similar stability as in whole blood and plasma matrices. The analyzed studies showed that storing the urine sample in frozen environments appears to be the most effective way to increase cannabinoid stability. Like in the instance of blood and plasma samples, cannabinoids’ adsorptive loss onto the containers’ surfaces also appears to be a problem with urine matrix, and therefore, the use of glass containers is recommended. In urine, adsorptive loss varies depending on urine sample pH, whereby a neutral/basic pH appears to lower the occurrence of this phenomenon. The addition of non-ionic surfactants to urine samples was shown to increase cannabinoid stability during the samples storing and handling.

Detection of intoxication and/or impairment caused by psychoactive substances like cannabinoids in oral fluid is becoming increasingly popular due to the ease of handling these samples. In oral fluid samples, similarly to blood and urine samples, THC concentrations tend to decrease over time, but the process can be significantly counteracted when the sample is refrigerated or, more effectively, frozen. Just like blood and urine, loss of cannabinoid concentration in oral fluids appears to be significantly greater if the samples are stored or handled using plastic-based tools; therefore, the use of glass vials and instruments is recommended. 

Lastly, due to the novelty of synthetic cannabinoids, research, particularly in the forensic field, has been looking at novel matrices that could contain detectable traces of cannabinoids/cannabinoid metabolites that would therefore indicate their consumption by individuals. Particular interest is placed on matrices such as breath, bile fluid, hair, sweat, cerumen, and dried blood spots. Although some of these matrices are effectively providing significant results, a lot more research is still required in this field.

## Figures and Tables

**Table 1:** Duration of storage stability for cannabinoids in blood based on temperature and collection container.

Matrix	T (°C)	Container	Stability	Note	Reference
Blood	−20 °C	Polystyrene plastic vials	60–100% loss between 4–24 weeks	Losses observed were 30–50% lower when stored in glass vials.	[11]
Blood	RT	Venoject tubes with rubber stoppers	2–8 weeks	At RT, THC concentrations significantly decreased after 2–8 weeks. Losses >90% after 6 months at RT.	[10]
Blood	RT	Green-top (Sodium heparin)	Stable for up to 1 week	THC-glu (ID) THC 1 week THCCOOH-glu < 1 week THCCOOH < 1 week 11-OH-THC < 1 week CBN 1 week CBD 1 week	[12]
Blood	4 °C	Green-top (Sodium heparin)	Stable for up to 6 months	THC-glu (ID) THC 3 months THCCOOH-glu 1 month THCCOOH 1 month 11-OH-THC 3 months CBN 6 months CBD (ID)	[12]
Blood	−20 °C	Green-top (Sodium heparin)	Stable for up to 6 months	THC-glu (ID) THC 3 months THCCOOH-glu 3 months THCCOOH 6 months 11-OH-THC 6 months CBN 3 months CBD (ID)	[12]
Blood	TR	Gray-top tubes (Sodium fluoride tubes)	Stable for up to 1 week	THC 1 week 11-OH-THC 1 week THCCOOH-glu < 1 week THCCOOH 1 week	[13]
Blood	4 °C	Gray-top tubes (Sodium fluoride tubes)	Stable for up to 6 months	THC 6 months 11-OH-THC 6 months THCCOOH-glu 1 week THCCOOH 6 months	[13]
Blood	−20 °C	Gray-top tubes (Sodium fluoride tubes)	Stable for up to 1 year	THC 6 months 11-OH-THC 1 year THCCOOH-glu3 months THCCOOH 1 year	[13]
Blood	RT	Green-top tubes (sodium heparin)	Stable for up to 1 week	THC 1 week 11-OH-THC 1 week THCCOOH-glu < 1 week THCCOOH < 1 week	[13]
Blood	4 °C	Green-top tubes (sodium heparin)	Stable for up to 3 months	THC 3 months 11-OH-THC 3 months THCCOOH-glu 1 month THCCOOH 1 month	[13]
Blood	−20 °C	Green-top tubes (sodium heparin)	Stable for up to 6 months	THC 3 months 11-OH-THC 6 months THCCOOH-glu 3 months THCCOOH 6 months	[13]
Blood (Synthetic Cannabinoids)	−20 °C	Glass Vials	Stable for up to 12 weeks	AB-Fubinaca, AB-Pinaca, UR-144 and XLR-11 remained stable for 12 weeks.	[2]
Blood (Synthetic Cannabinoids)	4 °C and RT	Glass Vials	Stable for up to 12 weeks	AB-Fubinaca, AB-Pinaca, and UR-144 remained stable for 12 weeks. XLR-11 significantly degraded by 31–73% after 3 weeks, and by 70–90% after 12 weeks.	[2]
Blood	−20 °C and 4 °C	Glass Vials	Stable for approximately 6 months	Loss of approximately 20% of their initial concentration.	[16]

RT: Room temperature; ID, inconclusive data (only 1 or 2 participant pools exceeded assay limit of quantification during baseline analysis).

**Table 2:** Duration of storage stability for cannabinoids in plasma based on temperature and collection container.

	T (°C)	Container	Stability	Note	Reference
Plasma	RT	Green-top (Sodium heparin)	Stable up to 1 week	THC-glu 1 week THC 1 week THCCOOH-glu < 1 week THCCOOH < 1 week 11-OH-THC < 1 week CBN 1 week CBD 1 week	[12]
Plasma	4 °C	Green-top (Sodium heparin)	Stable for up to 6 months	THC-glu 6 months THC 6 months THCCOOH-glu 2 weeks THCCOOH 2 weeks 11-OH-THC 6 months CBN 3 months CBD 6 months	[12]
Plasma	−20 °C	Green-top (Sodium heparin)	Stable for up to 1 year	THC-glu 1 year THC 1 year THCCOOH-glu 6 months THCCOOH 6 months 11-OH-THC 1 year CBN 1 year CBD 1 year	[12]
Plasma	RT	Gray-top tubes (Sodium fluoride tubes)	Stable for up to 1 week	THC 1 week 11-OH-THC 1 week THCCOOH-glu < 1 week THCCOOH < 1 week	[13]
Plasma	4 °C	Gray-top tubes (Sodium fluoride tubes)	Stable for up to 6 months	THC 3 months 11-OH-THC 6 months THCCOOH-glu 1 week THCCOOH 1 month	[13]
Plasma	−20 °C	Gray-top tubes (Sodium fluoride tubes)	Stable for up to 1 year	THC 1 year 11-OH-THC 1 year THCCOOH-glu 3 months THCCOOH 1 year	[13]
Plasma	RT	Green-top tubes (sodium heparin)	Stable for up to 1 week	THC 1 week 11-OH-THC < 1 week THCCOOH-glu < 1 week THCCOOH < 1 week	[13]
Plasma	4 °C	Green-top tubes (sodium heparin)	Stable for up to 6 months	THC 6 months 11-OH-THC 6 months THCCOOH-glu 2 weeks THCCOOH 2 weeks	[13]
Plasma	−20 °C	Green-top tubes (sodium heparin)	Stable for up to 1 year	THC 1 year 11-OH-THC 1 year THCCOOH-glu 6 months THCCOOH 6 months	[13]

RT: Room temperature.

**Table 3:** Duration of storage stability for cannabinoids in urine based on temperature and collection container.

Matrix	T (°C)	Container	Stability	Note	Reference
Urine	−20 °C	Glass vials and polyethylene plastic vials	Stable for approximately 20 weeks	85% recovery.	[21]
Urine	4° C and RT	Glass vials and polyethylene plastic vials	Not stable	At 4 °C and RT, in glass vials, recoveries were approximately 37% and 33%, respectively. In plastic vials, losses were 17% and 5% higher respectively.	[21]
Urine	−20 °C	Polypropylene container	Stable for 3 years	Maximum loss of 19.6 +/− 6.7% over a maximum time of 3 years.	[22]
Urine	−20 °C	Polypropylene container	Stable for 6 months	Remained stable for the whole duration of the experiments.	[23]
Urine	4 °C	Polyethylene and polypropylene plastic	n/a	Rapid loss was observed for both containers.	[25]
Urine	25 °C	Polyethylene plastic	Stable	No significant loss observed.	[25]
Urine	25 °C	Polypropylene plastic	Stable	Small loss (approximately 5%) was observed.	[25]
Urine	2–8 °C	High-density polyethylene nalgene containers	Minimal decrease in concentration over a 79-day period	(Approximately 11% loss)	[26]
Urine		Doxtech bottles with external barcodes		Loss of approximately 14% compared to the initial solution.	[28]
Urine		Doxtech bottles with internal barcodes		Loss of approximately 50% compared to the initial solution.	[28]
Urine	−20 °C	Pyrex bottles	Losses < 20% after 49 weeks	Solution treated with the surfactant Tergitol.	[29]
Urine	−20 °C	Pyrex bottles	Losses > 20% after 21 weeks	Untreated solution.	[29]

RT: Room temperature.

**Table 4:** Duration of storage stability for cannabinoids in oral fluids based on temperature and collection container.

	T (°C)	Container	Stability	Note	Reference
Oral Fluid	−20 °C, 4 °C, 21 °C	Polypropylene plastic	Experiment duration: 6 weeks	THC losses were reported to be 21% at −20 °C, 87% at 4 °C, and 86% at 21 °C.	[30]
Oral Fluid	4 °C and RT	Polypropylene plastic	Experiment duration: 6 days	The solution was treated with a 0.1 M phosphate buffer. Losses reported > 20%.	[30]
Oral Fluid	4 °C and RT	Glass vials	Experiment duration: 6 days	The solution was treated with a 0.1 M phosphate buffer. Losses reported < 10%.	[30]
Oral Fluid	RT	Polypropylene plastic tubes	Not stable	After 24 h, recoveries ranged between 29 and 65%. After 72 h, recoveries ranged between 9 and 54%.	[34]
Oral Fluid	4 °C	Polypropylene plastic tubes	Not stable	After 24 h, recoveries ranged between 83 and 103%. After 72 h, recoveries ranged between 75 and 79%.	[34]
Oral Fluid	RT and 4 °C	RapidEASE high-density borosilicate glass tubes	Stable	After 72 h at both temperature conditions, recoveries ranged between 84 and 114%.	[34]
Oral Fluid	RT and 4 °C	Polypropylene containers	Stable	Loss of 40–50% in both temperature conditions after 4 weeks.	[34]

RT: Room temperature.

## Abbreviations

Cannabinoids	
THC	Delta-9-Tetrahydrocannabinol
TCH-glu	THC-glucuronide
THCCOOH	11-nor-9-carboxy-THC
THCCOOH-glu	THCCOOH-glucoronide
11-OH-THC	11-Hydroxy-Delta-9-Tetrahydrocannabinol
CBN	Cannabinol
CBD	Cannabidiol
Synthetic Cannabinoids	
AB—Pinaca	
AB—Fubinaca	
XLR—11	
UR—144	
WIN 55,212-2	
Others	
CB1	Cannabinoid Receptor 1
CB2	Cannabinoid Receptor 2
ASC	Ascorbic Acid
FX	Fluoride Oxalate
